# SLR-superscaffolder: a de novo scaffolding tool for synthetic long reads using a top-to-bottom scheme

**DOI:** 10.1186/s12859-021-04081-z

**Published:** 2021-03-25

**Authors:** Lidong Guo, Mengyang Xu, Wenchao Wang, Shengqiang Gu, Xia Zhao, Fang Chen, Ou Wang, Xun Xu, Inge Seim, Guangyi Fan, Li Deng, Xin Liu

**Affiliations:** 1 BGI Education Center, University of Chinese Academy of Sciences, Shenzhen, 518083 China; 2 BGI-Qingdao, BGI-Shenzhen, Qingdao, 266555 China; 3 State Key Laboratory of Agricultural Genomics, BGI-Shenzhen, Shenzhen, 518083 China; 4 MGI, BGI-Shenzhen, Shenzhen, 518083 China; 5 BGI-Shenzhen, Shenzhen, 518083 China; 6 China National GeneBank, BGI-Shenzhen, Shenzhen, 518120 China; 7 Integrative Biology Laboratory, College of Life Sciences, Nanjing Normal University, Nanjing, 210046 China; 8 School of Biology and Environmental Science, Queensland University of Technology, Brisbane, 4000 Australia

**Keywords:** Genome assembly, Synthetic long reads, Next-generation sequencing, Scaffolding

## Abstract

**Background:**

Synthetic long reads (SLR) with long-range co-barcoding information are now widely applied in genomics research. Although several tools have been developed for each specific SLR technique, a robust standalone scaffolder with high efficiency is warranted for hybrid genome assembly.

**Results:**

In this work, we developed a standalone scaffolding tool, SLR-superscaffolder, to link together contigs in draft assemblies using co-barcoding and paired-end read information. Our top-to-bottom scheme first builds a global scaffold graph based on Jaccard Similarity to determine the order and orientation of contigs, and then locally improves the scaffolds with the aid of paired-end information. We also exploited a screening algorithm to reduce the negative effect of misassembled contigs in the input assembly. We applied SLR-superscaffolder to a human single tube long fragment read sequencing dataset and increased the scaffold NG50 of its corresponding draft assembly 1349 fold. Moreover, benchmarking on different input contigs showed that this approach overall outperformed existing SLR scaffolders, providing longer contiguity and fewer misassemblies, especially for short contigs assembled by next-generation sequencing data. The open-source code of SLR-superscaffolder is available at https://github.com/BGI-Qingdao/SLR-superscaffolder.

**Conclusions:**

SLR-superscaffolder can dramatically improve the contiguity of a draft assembly by integrating a hybrid assembly strategy.

**Supplementary Information:**

The online version contains supplementary material available at 10.1186/s12859-021-04081-z.

## Background

Synthetic long read (SLR) technologies [1–4], including single tube long fragment read (stLFR) sequencing [5], have recently been developed to allow co-barcoding of next-generation sequencing (NGS) short reads from the same long DNA fragment. Similar to the previous whole-genome shotgun sequencing strategy for BAC [6] or fosmid libraries [7], an SLR library can retain long-range genomic information but is more cost-effective. Since the relation between neighboring sequences is recoverable, based on the barcode shared by reads from the same DNA fragment, SLR data can be applied to haplotyping [1, 3, 4, 8, 9], structural variation detection [10–12] and de novo genome assembly [13–19].

Limited by the current SLR sequencing technologies, each DNA fragment cannot be directly reconstructed, as TruSPAdes does with a TruSeq dataset [20]. This is because the co-barcoding read coverage of a single DNA fragment is too low to satisfy the minimum assembly requirement. There are several genome assembly tools designed for each specific SLR library type. For contiguity preserving transposition sequencing (CPT-seq) reads, Adey and colleagues developed fragScaff to conduct scaffolding using the minimum spanning tree (MST) algorithm on a scaffold graph based on co-barcoding information [13]. Their results for the human genome show a greater improvement for input assemblies of high contiguity (NG50 ~ 100 kb). Kuleshov et al*.* utilized SLR sequencing technology from Illumina [2] and built scaffolds by combining co-barcoding with paired-end information to construct a scaffold graph and heuristically removing spurious edges using Architect [14]. For organisms with small genome sizes, the improvement by Architect also shows a clear dependence on the contiguity of the input assembly. ARCS [16] and ARKS [17] are developed by Warren et al*.* to use 10X Genomics Chromium data (10XG-linked reads) [4]. ARKS accelerates the scaffolding procedure and dramatically increases the NG50 of high-quality input assemblies (NG50 ~ 4.7 or 14.7 Mb). Weisenfeld et al*.* also developed a de novo assembler named Supernova for raw 10XG-linked reads [15]. Recently, based on analyzing the assembly graph, a universal assembler CloudSPAdes is developed by Tolstoganov et al. [19]. However, both Supernova and CloudSPAdes do not provide independent modules for scaffolding, and thus they cannot be combined with other sequencing data conveniently. For standalone scaffolders, an input assembly with long contiguity is usually required to obtain the co-barcoding information with sufficient completeness and accuracy to construct scaffolds efficiently. Thus, it is still a challenge to develop a robust scaffolder that is insensitive to input quality to effectively improve different draft assemblies.

Here, we presented a standalone scaffolder (SLR-superscaffolder) for stLFR reads. SLR-superscaffolder only requires a draft assembly (contigs or scaffolds) plus an SLR dataset as input. The tool exploited an overall top-to-bottom scheme, as outlined in Sect. 2.3, to hierarchically employ SLR information and lower the contiguity requirement of input assemblies. In addition, a screening algorithm was introduced in the ordering step to reduce the negative effect of non-ideal seed contigs on scaffolding.

We applied SLR-superscaffolder to an stLFR dataset for the human cell line NA12878 (HG001), benchmarked and compared with fragScaff, Architect, and ARKS. The results demonstrated that the scaffolds generated by SLR-superscaffolder had longer contiguity and higher accuracy than other tools for NGS-based draft assemblies. Since its algorithm was independent of the co-barcoding sequencing platform, SLR-superscaffolder had a great potential to be directly applied to various SLR datasets. The high robustness and accuracy would make SLR-superscaffolder a useful tool in a hybrid assembling strategy.

## Implementation

### A scaffolding model employing co-barcoding information

**Fig. 1:**
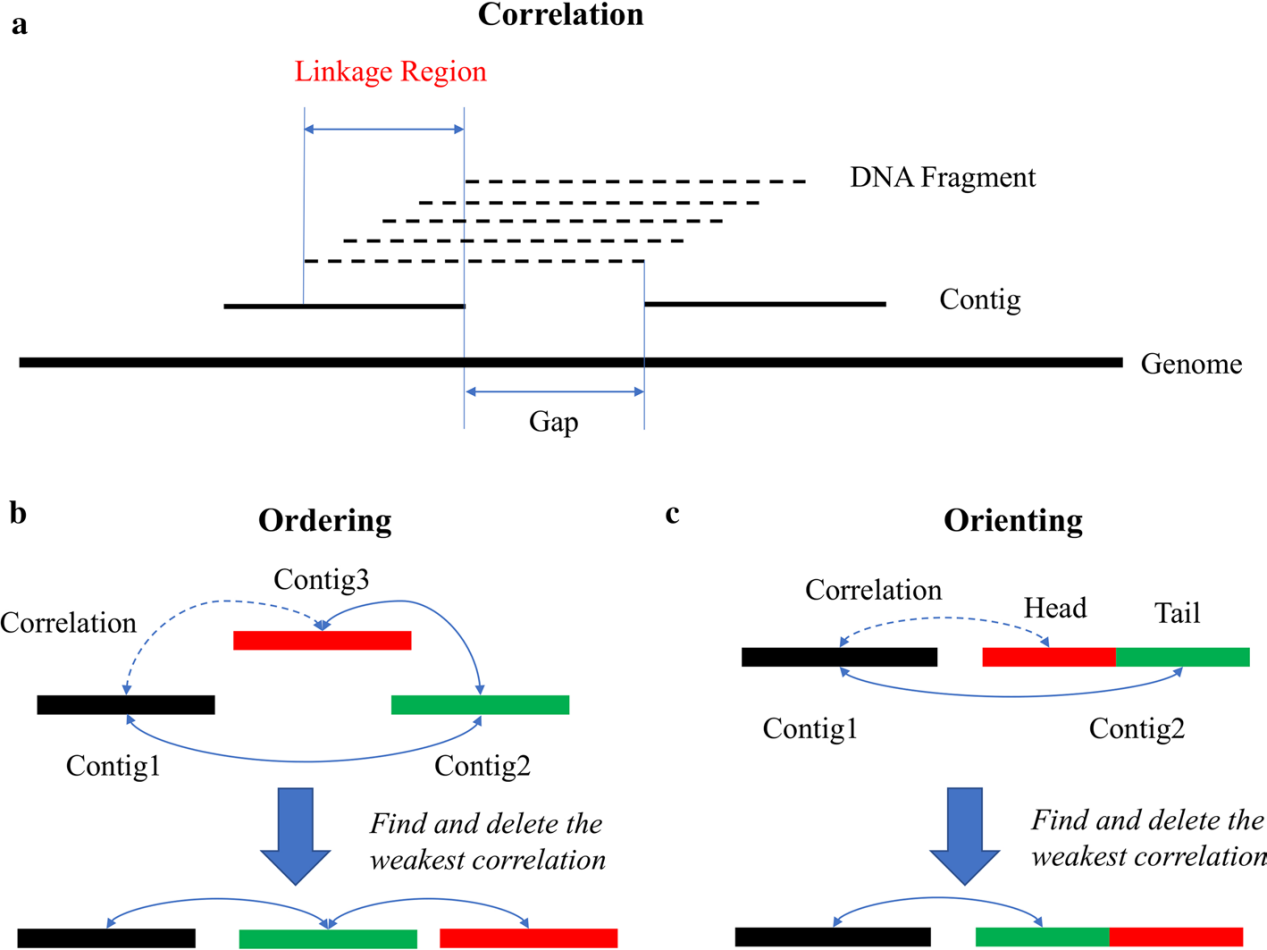
Scaffolding model of the co-barcoding correlation between two contigs (**a**), ordering of three contigs (**b**), and orienting of a contig using its neighboring contig with co-barcoding information (**c**)

Scaffolding is a process to determine the order and orientation of sequences by the correlations provided by different linkage information sources [21]. If the spatial relation is quantifiable, then the distance between two sequences can be estimated. SLR data contains two types of linkage information: paired-end and co-barcoding. Scaffolding based on paired-end information has been intensively discussed [22–24] and is not the focus of the present work. In SLR datasets, the co-barcoding information is obtained from shared barcodes (i.e., co-barcoded reads come from the same DNA fragment). As shown in Fig. 1a, the relation between adjacent contigs can be determined from mapped reads with the same barcode if these contigs overlap with the same DNA fragment. The length of the linkage region where the DNA fragment overlaps with both contigs is equal to the difference between DNA fragment length and gap size. Typically, the sequencing depth of DNA fragments is low, and thus not all the overlaps can be detected by the corresponding barcode. However, on the assumption that both the generation of DNA fragments and the capture of reads from each DNA fragment are unbiased and random, the linkage region length can be estimated by the correlation strength in statistics, which decreases with increasing gap size. This is the fundamental to order and orient contigs in the following steps (Fig. 1b, c). Figure 1b illustrates the ordering process of three adjacent contigs. The gap between Contig1 and Contig3 is the largest, indicating that their correlation strength is the weakest. Thus, the order of three contigs can be determined by deleting the weakest correlation in the graph. Since the linkage is undirected, a contig’s orientation cannot be determined straightforwardly. This problem can be transformed into the ordering procedure of three sub-contigs as shown in Fig. 1c, where Contig2 is split into two parts: the head and the tail.

### Quantified correlation strength

The correlation strength can be described by a function of shared barcodes between sequences. In this work, Jaccard Similarity (JS) was selected based on the discussion in Sect. 3.1. To avoid the effect of the contig length variation, JS between contig *m* and contig *n* was defined as the maximal JS between paired bins with the fixed size:

$$JS\left({contig}_{m},{contig}_{n}\right)=\mathrm{max}\left(JS\left({bin}_{i}^{m},{bin}_{j}^{n}\right)\right) for \, all\, pair(i,j)$$

where ${bin}_{i}^{m}$ is the *i*_*th*_ bin in contig *m*. The bins were chopped from both ends of a contig, and there was no gap or overlap between neighboring bins. JS between bins from different contigs can be calculated by

$$JS({bin}_{i}^{m},{bin}_{j}^{n})=\frac{|barcodes({bin}_{i}^{m})\cap barcodes({bin}_{j}^{n})|}{|barcodes({bin}_{i}^{m})\cup barcodes({bin}_{j}^{n})|}$$

where *barcodes*(${bin}_{i}^{m}$) was the set of barcodes whose corresponding reads were mapped to the ${bin}_{i}^{m}$.

### Overview of the algorithm and data preparation

SLR-superscaffolder was designed with a high degree of modularity. Overall, five modules were integrated: data preparation, ordering, orienting, local scaffolding, and gap size estimation, as shown in Additional file 1: Figure S1. Both paired-end and co-barcoding information of stLFR reads were used in scaffolding. To make efficient use of information with different correlation length scales, we adopted a top-to-bottom scheme, where the usage of global information occurs before local information. Specifically, the global scaffolding, including ordering and orienting using the co-barcoding information, occurs prior to paired-end-based local scaffolding. In the co-barcoding-based scaffolding, global ordering occurs prior to local orienting.

SLR-superscaffolder requires an SLR dataset plus a draft assembly as input. A draft assembly can be a set of contigs or scaffolds pre-assembled by various types of datasets (hereafter, we refer to contigs). Before scaffolding, we calculated the correlation between contigs to construct a scaffold graph and chose seed contigs to reduce the graph complexities caused by repeats. BWA (version 0.7.17) [25] was used to align stLFR reads to contigs, and reads only with a unique alignment were used to provide barcoding information based on their aligned positions on contigs. The ideal seed contigs are long and non-repetitive in the genome, without any misassemblies. Their mapped read depth should be around the average. As a result, the seed contigs were chosen according to a length threshold and an interval centered on the average depth. However, a few repetitive or misassembled contigs might be contained in seeds. Therefore, it is necessary to reduce the negative effect of these non-ideal seed contigs on scaffolding.

### Ordering

In our scheme, the order of contigs at a global scale was firstly determined by co-barcoding information. We constructed an undirected-weighted scaffold graph using JS between any two contigs. A node represents a seed contig. A weighted edge is created between two contigs when the JS between the contigs is higher than a given threshold; in that case, the weight of the edge is equal to the value of the JS between the corresponding contigs. The junction is a node with a degree more than two and the tip node is with a degree equal to one. The branch is a linear path from a tip node to the nearest junction. A branch with less than three nodes is defined as a tip branch, otherwise it is defined as a long branch. A junction with more than two long branches is defined as a long junction, otherwise it is defined as a tip branch. As described in Algorithm 1, the MST of a co-barcoding scaffold graph was obtained using Prim’s algorithm, and then the tip branches of the MST were pruned, finally the branches of the pruned MST were used to order the seed contigs. However, there are still too many junctions in a pruned MST to render the above process inefficient for ordering. We analyzed the property of contigs around junctions and found that long junctions strongly correlated with the non-ideal seed contigs, as discussed in Sect. 3.2. Thus, Algorithm 2 was designed to remove non-ideal seed contigs. The number of iterations and the ratio of screened contigs were set to avoid a possible significant reduction of connectivity in the co-barcoding scaffold graph.
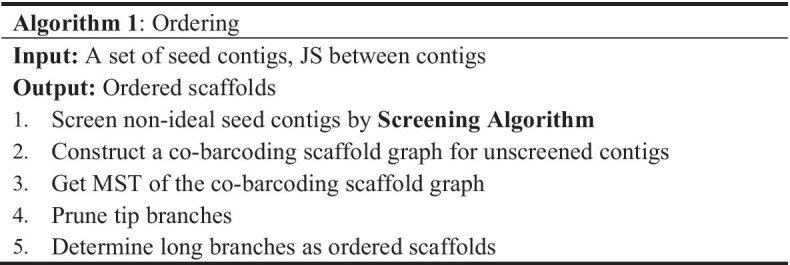

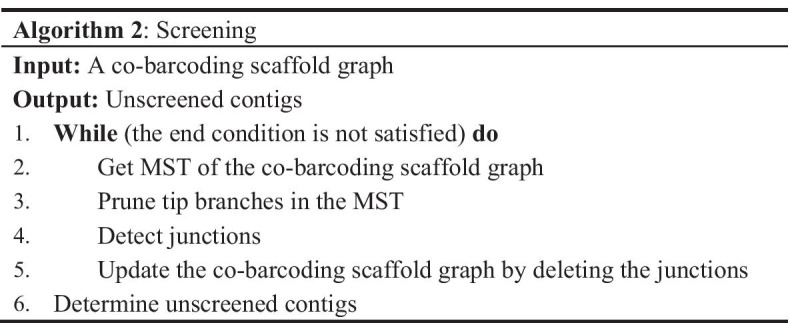


### Orienting

Because the co-barcoding information is undirected, it cannot be directly used for orienting. Thus, each contig was firstly split into two parts to be oriented, as shown in Fig. 1c. In this work, the head of a contig refers to the part from the 5′ terminal to the middle point and the residue is the tail. Unlike previous tools, which simultaneously determine order and orientation, we utilized the relation between neighboring contigs in an ordered scaffold to facilitate the orientation of each contig by the consensus strategy shown in Algorithm 3. In this strategy, each neighboring contig can provide a support for the contig’s orientation, as shown in Fig. 1c. The orientation of a contig has two states: an up state meaning the same direction relative to that of the ordered scaffold, and a down state meaning the opposite direction. The supported state was determined by the JS between the head and the neighboring contig (JS_Head) and that between the tail and the neighbor (JS_Tail). To increase computational efficiency, all neighboring contigs were uniformly split into two parts.
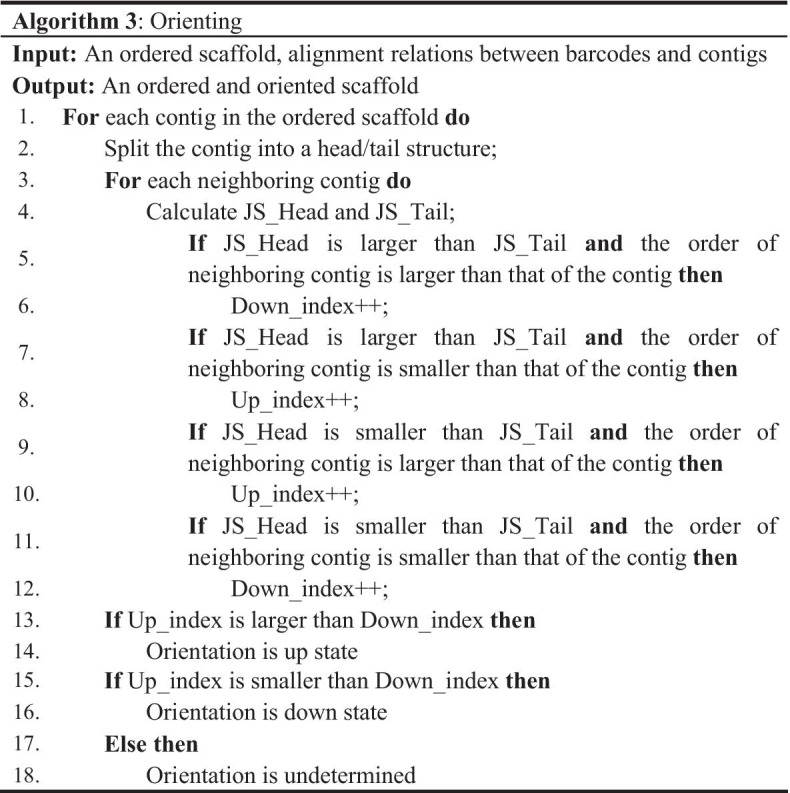


### Local scaffolding

In the above steps, most seed contigs have been ordered and oriented by the co-barcoding information. The unscaffolded contigs include non-seed contigs labeled in data preparation, those of tip branches in the MST, and those screened in the ordering step. These contigs can be further scaffolded by the local paired-end information of stLFR reads. In this step, we inserted the first two types of contigs into the gaps of oriented scaffolds according to Algorithm 4. To avoid the complex structures caused by repeat sequences on a global scale, only unscaffolded contigs with strong co-barcoding correlation to the paired contigs of a gap were clustered as candidates for local scaffolding. In the local directed paired-end scaffold graph, the nodes refer to candidate contigs of the gap, and the directed edges refer to connections verified by read pairs more than a threshold. The shortest connected path between the paired contigs was determined as the local scaffold using a depth-first search strategy.
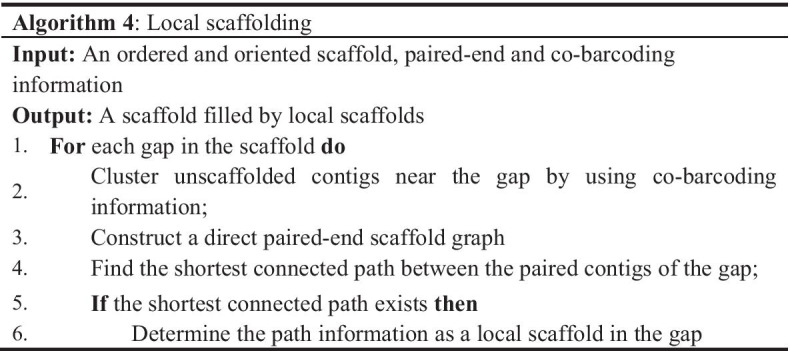


### Gap size estimation

We estimated the gap size between adjacent contigs in the ordered and oriented scaffolds, similar to the method in ARKS [17]. Specifically, gap size was determined by an empirical relation between the distance and the JS for gaps constructed by co-barcoding information (Additional file 1: Figure S1E). Although the exact distance between two reads with the same barcode is unknown, we observed a strong relation between JS and the distance of two sequences in human chromosome 19 (Chr19) (Additional file 1: Figure S2). Then we applied a linear fit using the least square method to obtain the correlation function for the gap size estimation step. The size was uniformly set to 11 bp for gaps constructed by the paired-end information due to the limited resolution.

### Evaluation

The standard metrics of QUAST (version 5.0.2) [26] were used to evaluate the efficiency and accuracy of assembled results, where Minimap2 [27] is used to get valid alignments. QUAST defines a major misassembly if an alignment difference is larger than 1 kb relative to the reference. These are further categorized into relocations, inversions, and translocations. An inversion indicates a reversion of part of a contig with respect to the reference genome. A relocation indicates a rearrangement of part of a contig within a chromosome. A translocation indicates a rearrangement of part of a contig between chromosomes. The relocations and translocations are used to measure ordering performance, while inversions are used for orienting in this work. The QUAST evaluations are run with default parameters, except for the lower contig length threshold (*-m 1000*).

The topological properties of an MST are changed by screening nodes in the ordering algorithm. To analyze the effects of the screening algorithm on the MST, the nodes were evaluated according to their topology in the graph as described in Sect. 2.4 and the edges were also evaluated by alignments of connected contigs against the reference. The edges were categorized into four classes: 1-order, 2-order, high-order, and error edges. If there are no contigs between a paired contigs in the reference genome, the correlation between the paired contigs was defined as a 1-order edge. If there is one, then the correlation was defined as 2-order. More middle contigs make the correlation a high-order edge. If the paired contigs are misassembled, then the correlation was defined as an error edge.

### Draft assemblies and datasets

In this work, three draft assemblies for HG001 were used as input, including the contigs assembled by MaSuRCA (version 3.3.5) [28] with 70 × stLFR reads only (MaSuRCA contigs), scaffolds assembled by SOAP*denovo2* (version r241) [29] with the same stLFR reads and an additional 20 × PE PCR-free NGS dataset (SOAP*denovo* scaffolds) and the contigs assembled by Canu (version 1.9) [30] with 30 × Oxford Nanopore technology (ONT) reads (ONT contigs) downloaded from Jain et al. work [31]. The evaluation for these input assemblies, the access information of these sequencing datasets, and the basic sequencing statistics of stLFR and PCR-free NGS reads were listed in Additional file 1: Tables S1–S3, respectively. The stLFR library was constructed using an MGIEasy stLFR Library Prep Kit and sequenced on a BGISEQ-500 instrument. Pair-end NGS reads with a ~ 390 bp insert size were randomly extracted from a PCR-free library constructed by an MGIEasy FS PCR-Free DNA Library Prep Set V1.0 (MGI, cat. No. 1000013455) and sequenced by an MGISEQ-2000 PE150 instrument. For parameter sweeps and data analysis, the stLFR reads from human Chr19 were extracted according to read alignments against the reference genome.

## Results and discussion

### stLFR read properties

**Fig. 2:**
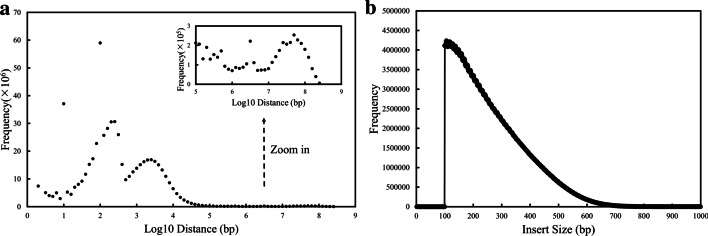
The distribution of distance between neighboring reads with the same barcode (**a**) and the insert size distribution of read pairs for stLFR reads (**b**)

For SLR datasets, the number of DNA fragments per barcode is an important property for downstream analyses [32], and ideally is one. To evaluate this property, we analyzed the distance distribution of neighboring reads with the same barcode from the same DNA fragment and those from different DNA fragments, as shown in Fig. 2. The distances were calculated after sorting aligned reads based on their genomic coordinates in the reference. There were three typical peaks, of which the third was expanded in the inset (Fig. 2a). The first peak corresponded to gaps between paired reads from the same short paired-end fragment, and its position was 251 bp. The second corresponded to gaps between neighboring reads from the same DNA fragment, and its position was about 2512 bp. The third corresponded to gaps between neighboring reads from different DNA fragments, and its position was 50 Mb. Compared with that of CPT-seq reads [13], the height ratio of the third peak to the second for stLFR reads was significantly lower, indicating that the average number of DNA fragments per barcode is less. This is consistent with a great number of barcodes for a typical stLFR library (50 million magnetic beads) compared with those of other SLR libraries. The insert size distribution of stLFR reads was non-Gaussian (Fig. 2b), different from a standard NGS library. The statistics demonstrate that the unique properties of stLFR reads require a more robust scaffolding algorithm to efficiently exploit the paired-end and co-barcoding information.

**Fig. 3:**
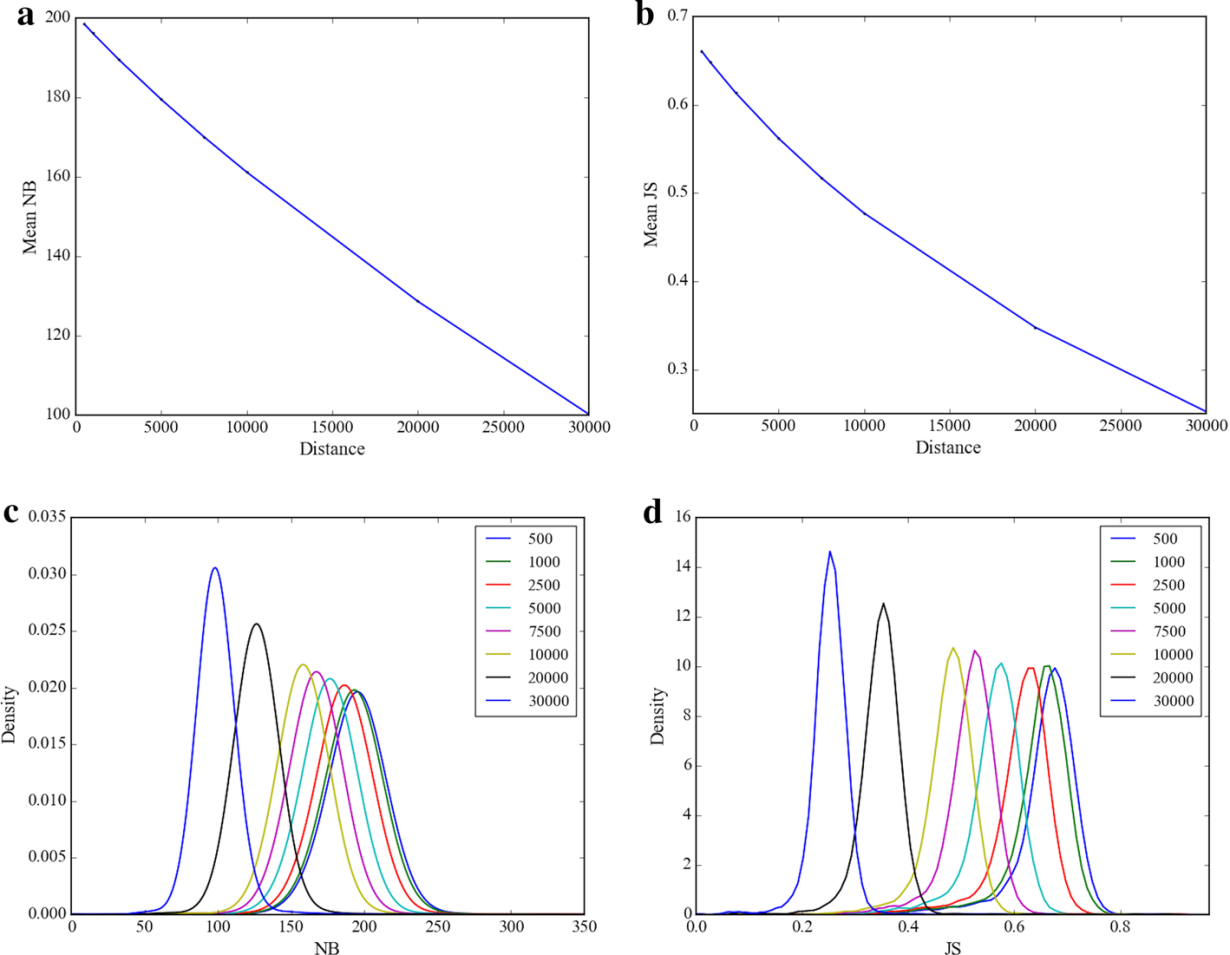
Mean values and distributions of NB and JS at different distances for a bin size of 5000 bp

A correlation of the co-barcoding information between contigs should be chose to construct the scaffold graph according to the scaffolding model. The number of shared barcodes (NB) between two contigs was used in fragScaff and ARKS. However, these tools ignored sequencing depth fluctuations of long DNA fragments randomly broken in SLR library. Instead, we used JS to reduce the effect of the fluctuation. To illustrate the advantage of JS relative to NB, all pairs of 5-kb bins in the human Chr19 reference were analyzed as a function of bin distance. Figure 3a, b showed that both JS and NB monotonically decreased as the bin distance increases. However, the monotonical decrease of JS was not observed for randomly barcoded reads (Additional file 1: Figure S2). These indicate that both JS and NB are valid to determine the order and orientation of contigs. As shown in Fig. 3c, d, the overlap between two normalized density distributions decreased as the bin distance increases for both JS and NB. However, the overlaps for NB were larger than those for JS. The same results were also observed in the distributions of different bin sizes, as shown in Additional file 1: Figures S3 and S4. Since the overlap is relevant to the error probability in scaffolding, these results indicate that JS is more efficient than NB.

### Assembly results using stLFR reads-alone

The MaSuRCA contigs were obtained by breaking the pre-assembled scaffolds at unknown bases (i.e., ‘N’). The evaluation of MaSuRCA contigs and run parameters were listed in Additional file 1: Tables S1 and S4. To evaluate the efficiency of SLR-superscaffolder (version 0.9), we benchmarked resulting scaffolds and compared them with those assembled by other SLR scaffolders, including fragScaff (version 140324.1), Architect (version 0.1), and ARKS (version 1.0.3). For each tool, run parameter sweeps were completed based on the human Chr19 dataset, and the optimal results were listed in Additional file 1: Table S5.

For MaSuRCA contigs, scaffolds assembled by SLR-superscaffolder showed the longest contiguity and the highest accuracy (Table 1). The scaffold NG50 was improved by about 1349 fold (from 13.1 kb to 17.6 Mb), while NGA50 was improved by about 29 fold (from 13.0 kb to 380.5 kb). Among other tools, fragScaff generated scaffolds with the highest quality; NG50 and NGA50 reached 400.9 kb and 17.5 kb, respectively. It is noted that the improvement by fragScaff, Architect, and ARKS were lower than those previously reported [17]. One possible reason is that the NG50 of MaSuRCA contigs is significantly shorter (~ 13 kb).

**Table 1:** Evaluation summary of assemblies using MaSuRCA contigs as input assemblies for HG001

	SLR-superscaffolder	fragScaff	Architect	ARKS	MaSuRCA
Number of scaffolds (> 1000 bp)	119,630	166,476	307,205	213,076	300,831
Largest scaffold (bp)	62,433,675	4,043,217	202,889	2,756,390	177,746
Total assembled length (bp)	3,345,341,888	3,672,256,418	3,038,310,109	2,908,519,565	2,907,642,015
NG50 (bp)	17,657,864	400,954	14,042	37,406	13,405
NGA50 (bp)	380,495	17,539	13,705	15,020	13,232
Relocation	11,015	92,267	3828	51,228	1648
Inversion	2939	5349	1637	5190	180
Translocation	2472	2294	903	10,828	849
Number of misassemblies	16,426	99,910	6368	67,246	4475

In our scheme, a screening algorithm was introduced to reduce the negative effect of non-ideal seed contigs in the ordering step. To evaluate its performance, the properties of MST were analyzed before and after screening based on the classification of nodes and edges defined in Sect. 2.8. According to the QUAST evaluation, there were 3083 non-ideal seed contigs out of 182,046 contigs in the initial MST. After screening, 2327 contigs were deleted, among which 858 were non-ideal seed contigs. It indicates that the screening algorithm can efficiently identify non-ideal seed contigs in an MST (Table 2).

**Table 2:** Statistics of nodes and edges in the MST before and after the screening

	Tip junction	Long junction	Tip node	Linear node	1-order edge	2-order edge	High-order edge	Error edge
Before screening	1826	354	2758	177,109	172,036	877	613	6441
After screening	1149	0	2430	176,140	172,016	865	432	3917

The initial MST contained 179,204 edges in total, and 96% were 1-order. It demonstrates the power of MST algorithm to determine 1-order edge. However, there were also 354 long junctions in the initial MST, which reduced the connectivity. Thus, the branches were too short to order the contigs efficiently. After the screening, the numbers of errors and higher-order edges were significantly reduced by 2524 and 181, but most of the 1-order and 2-order edges were maintained. Meanwhile, all the long junctions were removed. The screening algorithm reduced the MST complexity without weakening the capability to detect 1-order edges. Additional file 1: Tables S8 and S9 also showed the strong correlation of junctions and non-ideal seed contigs. 70.3% of long junctions were non-ideal seed contigs, and 88.4% of local graphs around long junctions contained at least one non-ideal seed contig.

### Effects of the length threshold of seed contigs

For the parameter optimization, we applied SLR-superscaffolder to human Chr19 stLFR reads. Compared with those of input contigs assembled by MaSuRCA, the scaffold NG50 and NGA50 values were improved by about 316 fold (from 27.5 kb to 8.7 Mb) and 33 fold (from 26.3 kb to 873.7 kb), with the optimized parameters and the seed length threshold of 7000 bp (Additional file 1: Table S6). In additional tests with simulated stLFR datasets, similar improvements were obtained for other model organisms with the same parameters (Additional file 1: Table S7). The methods of simulation and assembly were described in Additional file 1: Supplementary Note 1.

**Fig. 4:**
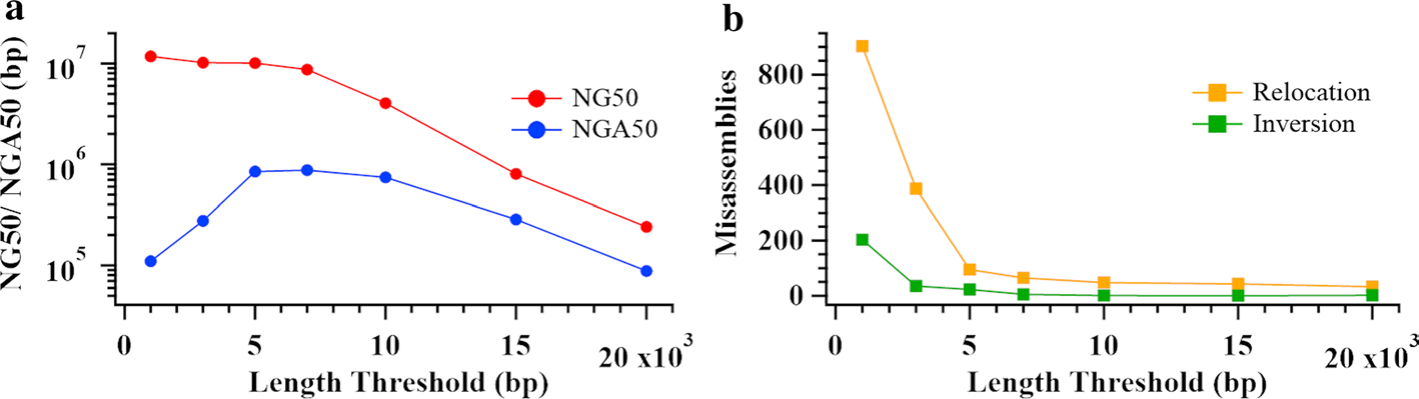
Quality of scaffolds assembled by SLR-superscaffolder with different seed contig length thresholds

SLR-superscaffolder’s parameters can be categorized into two groups: those dependent on stLFR read profiling, and those dependent on the contiguity and accuracy of input contigs. stLFR profiling is determined by the experimental processes, while the accuracy of input contig is unknown for a de novo assembly. Thus, only the effect of the input contigs’ contiguity was evaluated by varying the length threshold of seed contigs for the human Chr19 dataset. As shown in Fig. 4, with increasing length threshold, the scaffold NG50 monotonically decreased, while NGA50 reached a saturation peak between 5 and 10 kb. In terms of major misassemblies, the number of inversion and relocation errors monotonically decreased as the length threshold increases. It indicates that short seed contigs can enhance the contiguity of scaffolding results but introduce more misassemblies. Thus, it is imperative to balance the connectivity and complexity of a co-barcoding scaffold graph by tuning the number of short contigs. Although the balance is not determined only by the length threshold, the scaffold NGA50 saturation peak indicates that our tool can achieve a relatively optimal balance.

Effects of the local scaffolding by paired-end information were tested using the same dataset (Additional file 1: Table S6). Compared with those of without local scaffolding, the local scaffolding constructed 27 more linkages among the input contigs and generated scaffolds with 6 fewer inversions and 8 fewer relocations. The local scaffolding is an efficient way to use the complementarity between paired-end and co-barcoding information.

### Assembly results by combining stLFR with other sequencing reads

As a standalone scaffolding tool, SLR-superscaffolder can be easily implemented in a hybrid assembly strategy, where stLFR and other types of sequencing datasets can be used together. In this work, we also tested the hybrid assembling of a combination of stLFR and PCR-free NGS reads, as well as a combination of stLFR and ONT reads. In the first case, the input assembly (SOAP*denovo* scaffolds) consisted of scaffolds assembled by SOAP*denovo*2 with both stLFR and PCR-free NGS reads. In the second case, the input (ONT contigs) consisted of contigs assembled by Canu with ONT reads. The benchmarking results of different SLR scaffolders were listed in Table 3.

**Table 3:** Evaluation summary of assemblies using SOAP*denovo* scaffolds and ONT contigs as input for HG001

	SLR-superscaffolder	fragScaff	Architect	ARKS
Human (SOAP*denovo* scaffolds)				
Number of scaffolds (> 1000 bp)	48,278	54,193	79,435	80,400
Largest scaffold (bp)	35,605,665	59,127,605	796,758	10,897,000
Total assembled length (bp)	3,115,923,941	3,094,665,921	2,659,717,897	2,713,878,926
NG50 (bp)	9,113,260	2,346,521	54,245	468,461
NGA50 (bp)	1,510,911	101,813	44,836	59,899
Relocation	2373	32,588	1104	20,906
Inversion	105	1866	39	110
Translocation	3694	2926	875	4060
Number of misassemblies	6172	37,380	218	25,076
Human (ONT contigs)				
Number of scaffolds (> 1000 bp)	807	1051	1474	876
Largest scaffold (bp)	90,148,984	109,245,684	45,826,758	170,045,596
Total assembled length (bp)	2,829,830,390	2,828,106,943	2,823,722,824	2,823,836,148
NG50 (bp)	21,779,983	26,579,775	8,806,572	39,604,458
NGA50 (bp)	1,578,910	1,592,388	1,481,592	1,574,345
Relocation	4378	4182	3962	4266
Inversion	64	63	60	61
Translocation	1575	1453	1383	1607
Number of misassemblies	6017	5698	5405	5934

For SOAP*denovo* scaffolds, SLR-superscaffolder also obtained the longest contiguity and the highest accuracy. The scaffold NG50 was improved by 227 fold (from 40.1 kb to 9.1 Mb) and NGA50 by 44 fold (34.3 kb to 1.5 Mb). For ONT contigs, all scaffolders remarkably improved the contiguity, but not the accuracy. SLR-superscaffolder increased the NG50 from 6.6 Mb to 21.8 Mb (i.e., 3.3 fold), which was slightly less than ARKS (about sixfold) and fragScaff (about fourfold). The largest improvement of NGA50 was obtained using fragScaff, and SLR-superscaffolder had a comparable value. One of the problems is that the average number of misassemblies of an ONT contig is as high as 3.2, although its contig NG50 is large. These contigs with plenty of misassemblies were more likely to be screened, and thus the connectivity of the co-barcoding scaffold graph was significantly reduced. The above results indicate that the accuracy of input assemblies is important to conduct scaffolding with co-barcoding information.

### Overall performance

**Fig. 5:**
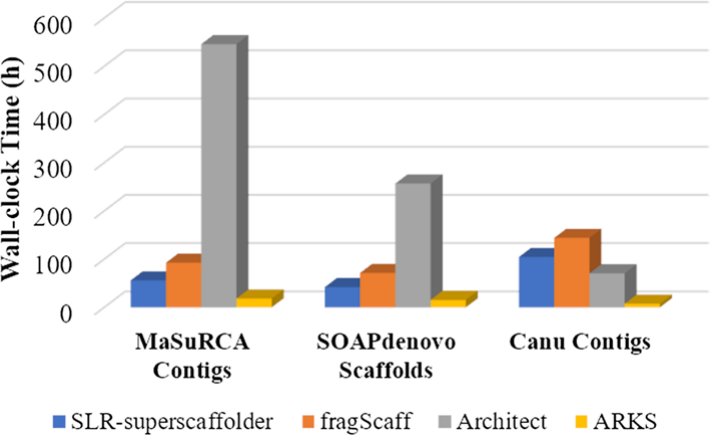
Histogram of time consumption of four scaffolders (SLR-superscaffolder, fragScaff, Architect, ARKS) for three input assemblies (MaSuRCA contigs, SOAP*denovo* scaffolds, Canu contigs)

We evaluated the running time of each scaffolder for three inputs on the same computational platform (Intel Xeon CPU E7-4890 v2 2.80 GHz, 60-core, 120 threads, and 3 Tb RAM), as shown in Fig. 5. All computations were limited to 20 threads. The results showed that ARKS had the best overall performance because it adopted a *k*-mer-based mapping strategy to avoid time-consuming pairwise aligning. SLR-superscaffolder ran approximately 1.5 fold and 4.3 fold faster than fragScaff and Architect, respectively. As listed in Additional file 1: Table S10, the data preparation step, including stLFR read mapping and co-barcoding information assignment, was the most time-consuming (averaging 58.3% of the total). The JS calculation was another time-consuming process, which could be reduced by random sampling of barcodes using the MinHash algorithm [33] (Additional file 1: Table S11). Note that we did not compare the peak memory consumption since the maximal usage depended on the aligners instead of the scaffolders themselves.

## Conclusions

stLFR sequencing data is a general SLR dataset with irregular insert size paired-end fragments and few DNA fragments per barcode. In this work, we have developed SLR-superscaffolder to employ stLFR co-barcoding information in de novo genome assembly with high efficiency. In our scheme, the use of co-barcoding information with long correlation length in global scaffolding is performed before using paired-end information in local scaffolding; and the global ordering step (with a lower input contig length requirement) is processed prior to the local orienting step. In our tests of the human genome, SLR-superscaffolder achieves several 100-fold scaffold NG50 improvements with high accuracy for input assemblies generated by NGS reads. These results demonstrate that the co-barcoding information from stLFR libraries can be used to significantly improve the quality of draft genomes in de novo assembly.

SLR-superscaffolder is the first SLR scaffolder that provides systematical screening of misassembled contigs to reduce the negative effect of these contigs in input assemblies. The strong correlation between misassembled contigs and long junctions in the MST of the scaffold graph is adopted to detect these contigs in our screening strategy. Compared with other SLR scaffolders, SLR-superscaffolder produces longer contiguity and higher accuracy for different input assemblies.

As a standalone scaffolder, SLR-superscaffolder improves the quality of assemblies generated by other types of libraries, such as standard NGS and single-molecule libraries. The co-barcoding information in other SLR libraries can also be exploited with appropriate format conversion, considering the general properties of the algorithm. Furthermore, since our approach is highly modularized, each step in SLR-superscaffolder can be separately combined with other types of sequencing datasets, such as single-molecule or mate-pair libraries, to design a new hybrid strategy in future.

### Availability and requirements

**Project name**: SLR-superscaffolder.

**Project home page**: https://github.com/BGI-Qingdao/SLR-superscaffolder

**Operating system**: Linux.

**Programming language**: C++

**Other requirements**: GCC (V4.8.3 or higher), BWA (V0.7.17), zlib.

**License**: General Public License V3.0

**Any restrictions to use by non-academics**: None.

## Supplementary Information

**Additional file 1.** **Table S1**. Summary of the input assemblies in this work. **Table S2**. Human genomic dataset sources. **Table S3**. Summary of human stLFR and NGS datasets used in this work. **Table S4**. Control parameters used in different scaffolders for different input assemblies. **Table S5**. Evaluation of human Chr19 assemblies based on MaSuRCA contigs by different scaffolders with the optimal parameters after the parameter sweeps. **Table S6**. Evaluation of human Chr19 assemblies for different tests. **Table S7**. Evaluation of SLR-superscaffolder’s scaffolding results for other model organisms using simulated stLFR data. **Table S8**. Statistics of tip and long junctions before and after conducting the screening algorithm. **Table S9**. Statistics of local properties of tip and long junctions before and after conducting the screening algorithm. **Table S10**. Runtime statistics for SLR-superscaffolder step by step. **Table S11**. Evaluation the MinHash strategy with different sample ratio for different organism genomes. **Figure S1**. The overall scheme of SLR-superscaffolder. **Figure S2**. Relations between Jaccard Similarity of barcodes and distance for two sequences in the reference for stLFR reads and randomly barcoded reads. **Figure S3**. Mean values and distributions of NB and JS at different distances for a bin size of 1,200 bp. **Figure S4**. Mean values and distributions of NB and JS at different distances for a bin size of 20,000 bp. **Supplementary Note 1**. Detailed test information for four model organisms.

## Data Availability

The source codes and instructions of SLR-superscaffolder are freely available on GitHub (https://github.com/BGI-Qingdao/SLR-superscaffolder, licensed under GNU General Public License V3.0). The stLFR dataset of HG001 is available on CNSA of CNGBdb (Access ID CNP0000066). The PCR-free NGS dataset of HG001 is available on CNSA of CNGBdb (Access ID CNP0000602). The ONT Canu assembly of HG001 is available at: https://ftp.ncbi.nlm.nih.gov/genomes/all/GCA/900/232/925/GCA_900232925.2_NA127878-rel5/GCA_900232925.2_NA127878-rel5_genomic.fna.gz.

## Abbreviations

SLR: Synthetic long reads
stLFR: Single tube long fragment reads
NGS: Next generation sequencing
CPT-seq: Contiguity preserving transposition sequencing
MST: Minimum spanning tree
10XG-linked reads: 10X genomics chromium technology
HG001: The human whole genome for cell line NA12878
JS: Jaccard Similarity
NB: Number of shared barcodes
Chr19: Chromosome 19
