# Supplementary material for: SLR-superscaffolder: a de novo scaffolding tool for synthetic long reads using a top-to-bottom scheme
Source: BMC Bioinformatics. 2021 Mar 25;22:158. doi: 10.1186/s12859-021-04081-z (PMC7993450; doi:10.1186/s12859-021-04081-z)
Supplement: Supplementary file 1 — Additional file 1. Table S1. Summary of the input assemblies in this work. Table S2. Human genomic dataset sources. Table S3. Summary of human stLFR and NGS datasets used in this work. Table S4. Control parameters used in different scaffolders for different input assemblies. Table S5. Evaluation of human Chr19 assemblies based on MaSuRCA contigs by different scaffolders with the optimal parameters after the parameter sweeps. Table S6. Evaluation of human Chr19 assemblies for different tests. Table S7. Evaluation of SLR-superscaffolder’s scaffolding results for other model organisms using simulated stLFR data. Table S8. Statistics of tip and long junctions before and after conducting the screening algorithm. Table S9. Statistics of local properties of tip and long junctions before and after conducting the screening algorithm. Table S10. Runtime statistics for SLR-superscaffolder step by step. Table S11. Evaluation the MinHash strategy with different sample ratio for different organism genomes. Figure S1. The overall scheme of SLR-superscaffolder. Figure S2. Relations between Jaccard Similarity of barcodes and distance for two sequences in the reference for stLFR reads and randomly barcoded reads. Figure S3. Mean values and distributions of NB and JS at different distances for a bin size of 1,200 bp. Figure S4. Mean values and distributions of NB and JS at different distances for a bin size of 20,000 bp. Supplementary Note 1. Detailed test information for four model organisms. [file 12859_2021_4081_MOESM1_ESM.docx]

**Supplementary Information**

**SLR-superscaffolder: a de novo scaffolding tool for synthetic long reads using a top-to-bottom scheme**

Lidong Guo*^a,b,c#^*, Mengyang Xu*^b,c,e,f#^*, Wenchao Wang*^b^*, Shengqiang Gu*^a^*, Xia Zhao*^d^*, Fang Chen*^d^*, Ou Wang*^e,f^*, Xun Xu*^e,f^*, Inge Seim*^g,h^*, Guangyi Fan*^b,c,e,f^*, Li Deng *^b,c,e,f*^* and Xin Liu*^b,c,e,f*^*

*^a^*BGI Education Center, University of Chinese Academy of Sciences, Shenzhen 518083, China

*^b^*BGI-Qingdao, BGI-Shenzhen, Qingdao 266555, China

*^c^*State Key Laboratory of Agricultural Genomics, BGI-Shenzhen, Shenzhen 518083, China

*^d^*MGI, BGI-Shenzhen, Shenzhen 518083, China

*^e^*BGI-Shenzhen, Shenzhen 518083, China

*^f^*China National GeneBank, BGI-Shenzhen, Shenzhen 518120, China

*^g^*Integrative Biology Laboratory, College of Life Sciences, Nanjing Normal University, Nanjing 210046, China

*^h^*School of Biology and Environmental Science, Queensland University of Technology, Brisbane 4000, Australia

*^*^*Corresponding authors:

Xin Liu ([liuxin@genomics.cn](mailto:liuxin@genomics.cn)),

Li Deng([dengli1@genomics.cn](mailto:dengli1@genomics.cn))

**^#^**These authors contributed equally to this work.

Table S1. Summary of the input assemblies in this work.

|  | Datasets | Number of scaffolds (>1,000 bp) | Largest scaffold | Total assembled length | NG50 (bp) | NGA50 (bp) | Relocation | Inversion | Number of misassemblies |
| --- | --- | --- | --- | --- | --- | --- | --- | --- | --- |
| Human Chr19 | MaSuRCA contigs | 4,083 | 148,918 | 57,024,748 | 25,956 | 25,384 | 114 | 4 | 118 |
| Human whole- genome | MaSuRCA contigs | 308,823 | 160,048 | 2,905,719,951 | 13,087 | 12,992 | 1,648 | 180 | 2,677 |
|  | SOAP*denovo2* scaffolds | 122,465 | 528,666 | 2,862,817,877 | 40,154 | 34,369 | 982 | 28 | 2,745 |
|  | ONT contigs | 1,685 | 38,914,416 | 2,823,828,058 | 6,593,913 | 1,403,850 | 3,944 | 60 | 5,364 |

Note: MaSuRCA run with parameters *GRAPH_KMER_SIZE = 63, cgwErrorRate=0.15*. SOAP*denovo2* run with parameters *-K 49, -p 16, -a 700, -d 1*.

Table S2. Human genomic dataset sources.

| Species | Datatype | Source |
| --- | --- | --- |
| *H. sapiens*  NA12878 | stLFR Dataset | https://ftp.cngb.org/pub/CNSA/data1/CNP0000066/CNS0007594/CNX0005851/CNR0006062 |
| *H. sapiens*  NA12878 | PCR-free NGS Dataset | https://ftp.cngb.org/pub/CNSA/data2/CNP0000602/CNS0106271/CNX0086627/CNR0106776 |
| *H. sapiens*  NA12878 | ONT Canu assembly | https://ftp.ncbi.nlm.nih.gov/genomes/all/GCA/900/232/925/GCA_900232925.2_NA127878-rel5/GCA_900232925.2_NA127878-rel5_genomic.fna.gz |
| *H. sapiens*  NA12878 | Reference Genome | ftp://ftp.ncbi.nlm.nih.gov/genomes/all/GCF/000/001/405/GCF_000001405.39_GRCh38.p13/GCF_000001405.39_GRCh38.p13_genomic.fna.gz |

Table S3. Summary of human stLFR and NGS datasets used in this work.

|  | Number of Read pairs | Average insert size(bp) | Length of read pair (bp) | Coverage | Number of Barcode |
| --- | --- | --- | --- | --- | --- |
| stLFR dataset of NA12878 chr19 | 21,507,205 | 170 | 100 | 73.4 | 1,105,848 |
| stLFR dataset of NA12878 whole genome sequences (WGS) | 1,035,984,388 | 229 | 100 | 64.6 | 37,196,043 |
| NGS data sets of NA12878 WGS | 479,007,051 | 390 | 150 | 47.9 | 0 |

Table S4. Control parameters used in different scaffolders for different input assemblies.

|  | Chr19  MaSuRCA Contig | Human NA12878  MaSuRCA Contig | Human NA12878  SOAP*denovo2* Scaffold | Human NA12878  ONT Contig |
| --- | --- | --- | --- | --- |
| SLR-superscaffolder | *-MB 7000, -HB 3500, -T 0.1, -P 200* | *-MB 7000, -HB 3500, -T 0.1, -P 1000* | *-MB 7000, -HB 3500, -T 0.1, -P 300* | *-MB 7000, -HB 3500, -T 0.1, -P 200* |
| fragScaff | *-E 20000, -j 5, -u 2, -p=A* | *-E 20000, -j 5, -u 2, -p=A* | *-E 20000, -j 5, -u 2, -p=A* | *-E 20000, -j 5, -u 2, -p=A* |
| Architect | *-T 3, -E 0.15, -P 0.1* | *-T 3, -E 0.15, -P 0.1* | *-T 3, -E 0.15, -P 0.1* | *-T 3, -E 0.15, -P 0.1* |
| ARKS | *-k 100, -a 1.0* | *-k 30, -a 1.0* | *-k 30, -a 1.0* | *-k 30, -a 1.0* |

Table S5. Evaluation of human Chr19 assemblies based on MaSuRCA contigs by different scaffolders with the optimal parameters after the parameter sweeps. Scaffolding of the draft assembly was performed with SLR-superscaffolder (*MST_BIN_SIZE*, *HT_BIN_SIZE*, *CLUSTER* and *PE_SEED_MIN* abbreviated to “*MB*”, “*HB*”, “*T*” and “*P*” respectively), fragScaff (*-m 1 -C 10 -t 8*), Architect (*--rc-abs-thr, --rc-rel-edge-thr and --rc-rel-prun-thr* abbreviated to “*T*”, “*E*” and “*P*” respectively), and ARKS (*-t 8 -c 5 -j 0.5 -z 3000 -e 30000 -m 50-6000 -r 0.05*).

Note: The runtime for MaSuRCA is the total assembly time, from raw reads to final scaffolds.

|  | Run parameters | Number of scaffolds(>1,000bp) | Largest scaffold (bp) | Total assembled length (bp) | NG50 (bp) | NGA50 (bp) | Relocation | Inversion | Number of misassemblies | Time | Peak Memory |
| --- | --- | --- | --- | --- | --- | --- | --- | --- | --- | --- | --- |
| SLR-superscaffolder | *-MB 7000, -HB 3500, -T 0.1, -P 200* | 1,461 | 13,595,631 | 62,941,506 | 8,696,596 | 873,719 | 160 | 9 | 169 | 56m | 1.51GB |
| fragScaff | *-E 20000, -j 5, -u 2, -p=A* | 1,611 | 1,789,170 | 73,536,076 | 343,719 | 25,384 | 2,264 | 4 | 2,268 | 4h54m | 1.42GB |
| Architect | *-T 3, -E 0.15, -P 0.1* | 3,286 | 179,888 | 57,015,198 | 29,949 | 25,783 | 465 | 4 | 469 | 4h49m | 1.42GB |
| ARKS | *-k 100, -a 1.0* | 2,596 | 2,452,186 | 56,780,331 | 196,104 | 33,812 | 749 | 28 | 777 | 17m | 4.98GB |
| MaSuRCA | *GRAPH_KMER_SIZE = 63, cgwErrorRate=0.15* | 3,499 | 148,918 | 57,069,141 | 27,512 | 26,350 | 157 | 5 | 162 | 3h28m | 17.02GB |

Table S6. Evaluation of human Chr19 assemblies for different tests. All parameters are optimized and set to default value, except the seed contig length thresholds and on-off of local scaffolding.

|  |  | Number of scaffolds (>1000 bp) | Largest scaffold (bp) | Total assembled length (bp) | NG50 (bp) | NGA50 (bp) | Relocation | Inversion | Number of misassemblies |
| --- | --- | --- | --- | --- | --- | --- | --- | --- | --- |
| MaSuRCA |  | 3,779 | 148,918 | 57,069,141 | 27,512 | 26,350 | 157 | 5 | 162 |
| SLR-superscaffolder |  | 2,186 | 13,595,631 | 62,941,506 | 8,696,596 | 873,719 | 160 | 9 | 169 |
|  | No local scaffolding | 2,213 | 13,621,100 | 63,116,164 | 8,706,614 | 850,387 | 168 | 15 | 183 |
| SLR-superscaffolder (with different length thresholds bp) | 1,000 | 1,329 | 23,756,072 | 76,490,713 | 11,754,257 | 110,175 | 990 | 207 | 1197 |
|  | 3,000 | 1,491 | 22,236,846 | 65,689,522 | 10,200,611 | 276,992 | 479 | 40 | 519 |
|  | 5,000 | 1,900 | 22,599,625 | 62,039,221 | 10,076,573 | 847,484 | 189 | 27 | 216 |
|  | 7,000 | 2,186 | 13,595,631 | 62,941,506 | 8,696,596 | 873,719 | 160 | 9 | 169 |
|  | 10,000 | 1,920 | 11,703,047 | 64,839,557 | 4,047,100 | 740,961 | 144 | 6 | 150 |
|  | 15,000 | 3,218 | 2,970,432 | 65,941,210 | 803,638 | 286,442 | 137 | 4 | 141 |
|  | 20,000 | 3,634 | 1,944,702 | 63,349,858 | 241,262 | 88,273 | 125 | 6 | 131 |

Table S7. Evaluation of SLR-superscaffolder’s scaffolding results for other model organisms using simulated stLFR data.

| Organisms | Assembly | Number of scaffolds (>1000 bp) | Largest scaffold (bp) | Total assembled length (bp) | NG50 (bp) | NGA50 (bp) | Relocation | Inversion | Translocation | Number of misassemblies |
| --- | --- | --- | --- | --- | --- | --- | --- | --- | --- | --- |
| *E. coli* | MaSuRCA contigs | 81 | 420,784 | 4,722,198 | 130,315 | 130,315 | 2 | 0 | 0 | 2 |
|  | SLR-superscaffolder scaffolds | 23 | 2,648,786 | 4,879,712 | 2,648,786 | 948,134 | 9 | 0 | 0 | 9 |
| *S. cerevisiae* | SOAP*denovo2* contigs | 522 | 147,768 | 11,270,032 | 38,082 | 38,082 | 0 | 0 | 0 | 0 |
|  | SLR-superscaffolder scaffolds | 158 | 1,500,213 | 11,951,521 | 778,106 | 685,155 | 9 | 0 | 0 | 9 |
| *C. elegans* | MaSuRCA contigs | 2,786 | 778,196 | 98,304,795 | 93,273 | 87,118 | 412 | 16 | 121 | 549 |
|  | SLR-superscaffolder scaffolds | 996 | 6,380,526 | 101,649,079 | 1,729,104 | 315,740 | 647 | 40 | 160 | 847 |
| *A. thaliana* | SOAP*denovo2* contigs | 9,170 | 188,650 | 105,360,744 | 23,163 | 23,163 | 1 | 0 | 0 | 1 |
|  | SLR-superscaffolder scaffolds | 4,620 | 12,620,129 | 120,059,064 | 7,334,765 | 5,556,282 | 10 | 1 | 0 | 11 |

Table S8. Statistics of tip and long junctions before and after conducting the screening algorithm.

|  | Tip junction | | Long junction | |
| --- | --- | --- | --- | --- |
| For the node | Unique | Non-unique | Unique | Non-unique |
| Before | 1,375 | 451 | 105 | 249 |
| After | 1,049 | 100 | 0 | 0 |

Table S9. Statistics of local properties of tip and long junctions before and after conducting the screening algorithm.

|  | Tip junction | | Long junction | |
| --- | --- | --- | --- | --- |
| For local graph of the node | Unique | Non-unique | Unique | Non-unique |
| Before | 1,045 | 781 | 41 | 313 |
| After | 917 | 232 | 0 | 0 |

Table S10. Runtime statistics for SLR-superscaffolder step by step.

|  | MaSuRCA contigs | | SOAP*denovo* scaffolds | | ONT contigs | |
| --- | --- | --- | --- | --- | --- | --- |
|  | Wall-clock time(h) | Percentage (%) | Wall-clock time (h) | Percentage (%) | Wall-clock time(h) | Percentage (%) |
| Total | 56.1 | 100.0 | 42.1 | 100.0 | 104.6 | 100.0 |
| Data preparation | 31.2 | 55.6 | 33.4 | 79.4 | 41.8 | 39.9 |
| Ordering | 12.0 | 21.4 | 2.5 | 6.0 | 1.8 | 1.7 |
| Orienting | 7.8 | 13.9 | 1.1 | 2.7 | 1.2 | 1.2 |
| Local scaffolding | 1.7 | 3.1 | 2.7 | 6.4 | 1.4 | 1.3 |
| Gap size estimation | 3.4 | 6.1 | 2.3 | 5.6 | 58.5 | 55.9 |

Table S11. Evaluation the MinHash strategy with different sample ratio for different organism genomes. All contigs used in tests were assembled by MaSuRCA.

|  | Sampling ratio | CPU time for calculating Jaccard Similarity (s) | Number of scaffolds (>1,000 bp) | Largest scaffold (bp) | Total assembled length (bp) | NG50 (bp) | NGA50 (bp) | Number of misassemblies |
| --- | --- | --- | --- | --- | --- | --- | --- | --- |
| *E.coli*  ( 100× simulated reads ) | 1.00 | 29 | 23 | 2,648,786 | 4,879,712 | 2,648,786 | 948,134 | 9 |
|  | 0.20 | 4 | 25 | 2,588,543 | 4,850,645 | 2,588,543 | 948,144 | 8 |
| *C. elegans*  ( 100× simulated reads ) | 1.00 | 899 | 996 | 6,380,526 | 101,649,079 | 1,729,104 | 315,740 | 847 |
|  | 0.20 | 123 | 983 | 5,134,315 | 101,655,582 | 1,776,398 | 307,990 | 871 |
|  | 0.10 | 46 | 993 | 5,138,943 | 101,613,064 | 1,773,476 | 278,722 | 899 |
| chr19  ( 70× stLFR reads ) | 1.00 | 304 | 1,461 | 13,595,631 | 62,941,506 | 7,038,144 | 873,719 | 169 |
|  | 0.40 | 84 | 1,475 | 12,180,130 | 64,234,952 | 7,123,147 | 715,205 | 202 |
|  | 0.30 | 59 | 1,482 | 12,260,074 | 64,065,788 | 4,341,313 | 672,778 | 235 |
|  | 0.20 | 36 | 1,502 | 9,528,684 | 63,718,042 | 4,006,740 | 438,703 | 271 |


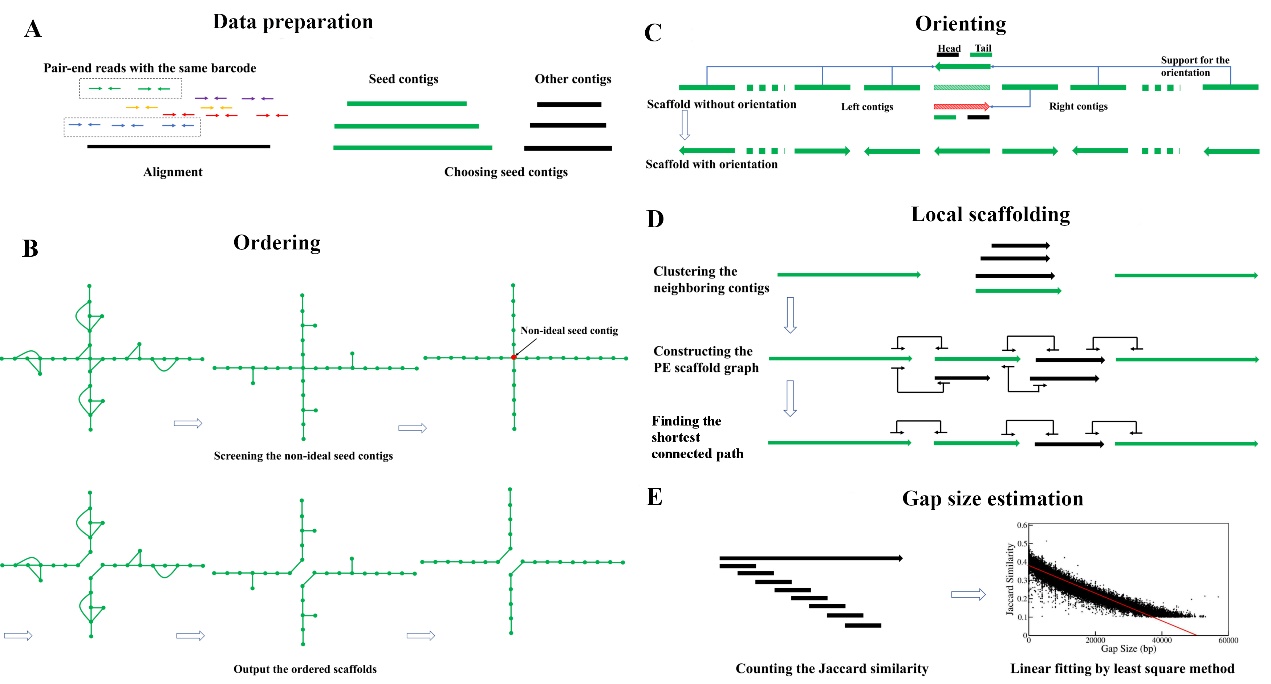


Figure S1. The overall scheme of SLR-superscaffolder. (A) In data preparation two sub-processes are included: aligning stLFR reads to the draft assembly and choosing seed contigs. (B) In ordering, non-ideal seed contigs are interactively screened, as shown in the upper three figures, and then ordered scaffolds are generated as shown in the lower three figures. (C) In orienting, n*_th_*-order neighboring contigs in each ordered scaffold determine the orientation state of each contig. (D) In local scaffolding, contigs near paired neighboring contigs in the scaffold are firstly clustered by co-barcoding information, and then a scaffold graph is further constructed by PE information. Finally, the shortest path among the neighboring contigs is determined as local scaffolds. (E) In gap size estimation, a statistical relation between Jaccard similarity of shared barcodes and distance is calculated for long contigs, and then an approximately linear relation is fitted using the least square method.


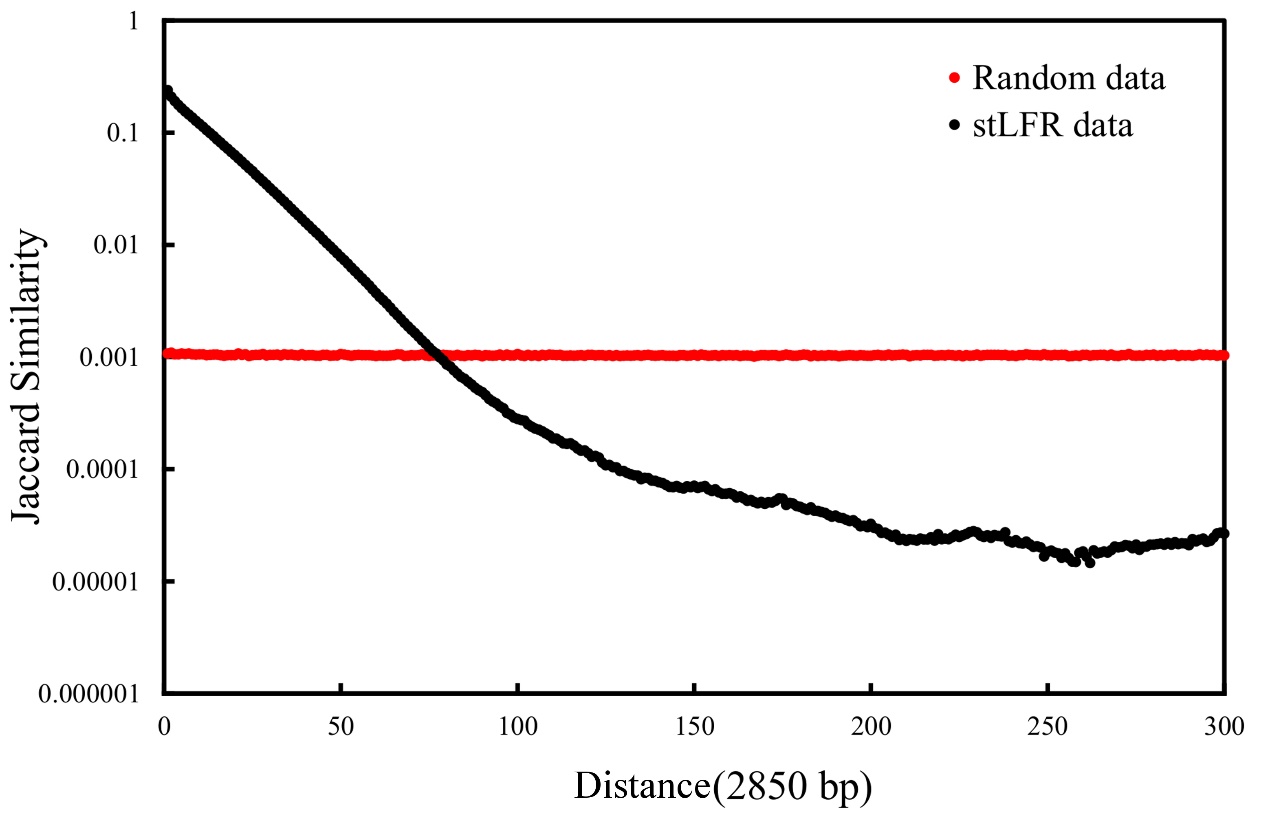


Figure S2. Relations between Jaccard Similarity of barcodes and distance for two sequences in the reference for stLFR reads and randomly barcoded reads. The randomly barcoded reads are generated by shuffling stLFR barcodes for the same read sets.


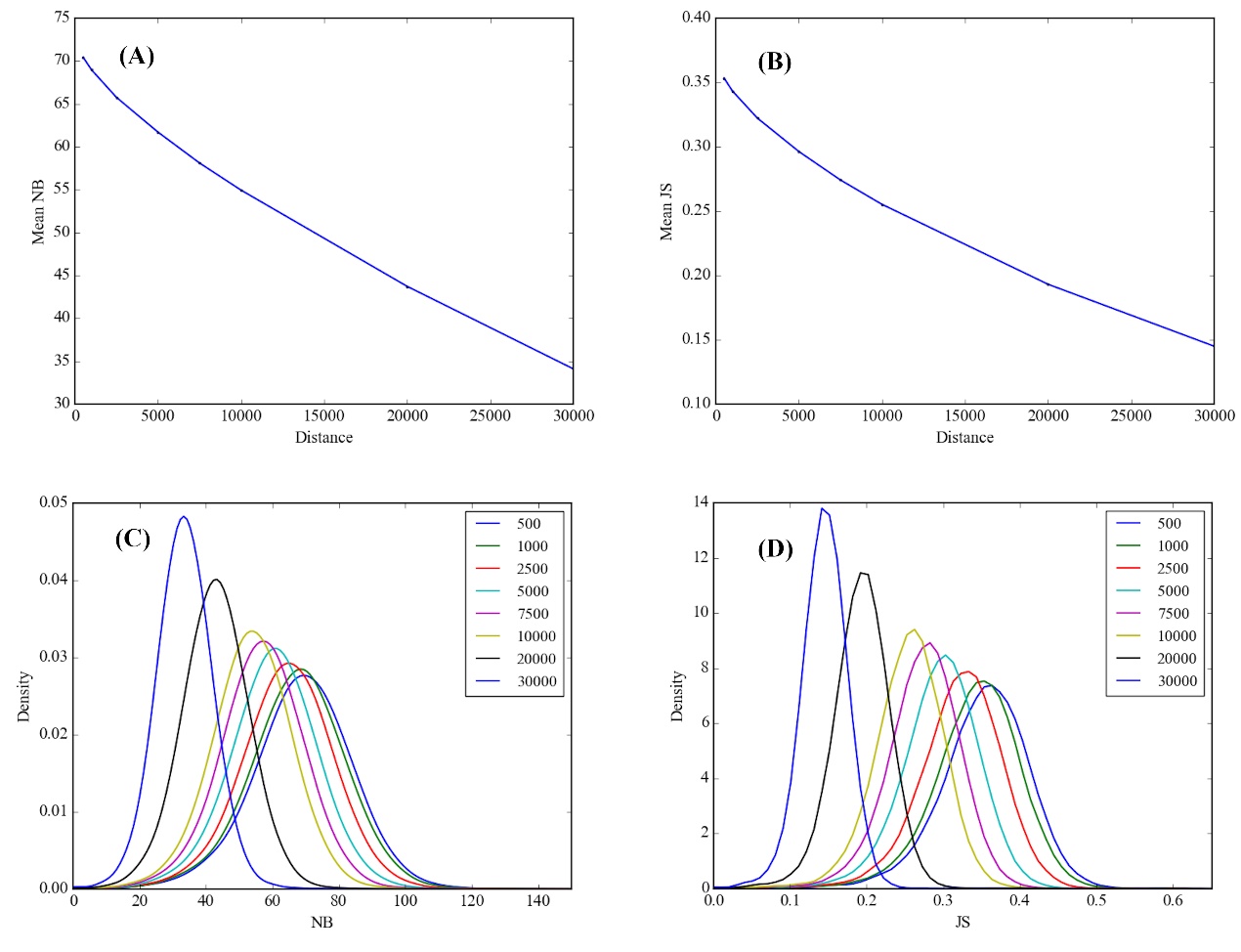


Figure S3. Mean values and distributions of NB and JS at different distances for a bin size of 1,200 bp. NB denotes the number of shared barcodes; JS, Jaccard Similarity.


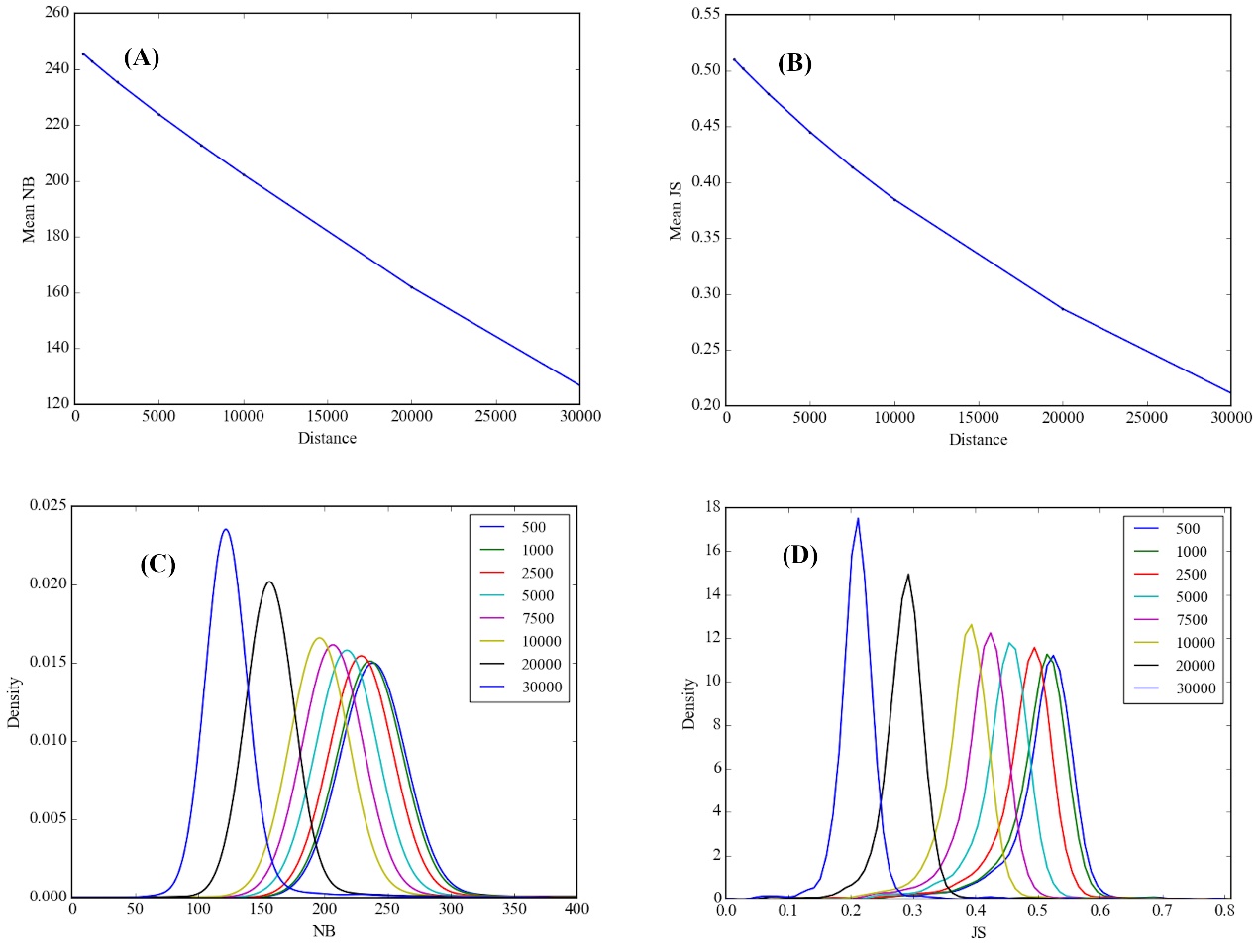


Figure S4. Mean values and distributions of NB and JS at different distances for a bin size of 20,000 bp. NB denotes the number of shared barcodes; JS, Jaccard Similarity.

**Supplementary Note 1**: Detailed test information for four model organisms

To investigate the effect of scaffolding, we also applied SLR-Superscaffolder to four model organisms with genome sizes ranging from 4 Mb to 120 Mb: a bacterium (*Escherichia coli*), a yeast (*Saccharomyces cerevisiae*), an invertebrate (*Caenorhabditis elegans*), and a plant (*Arabidopsis thaliana*). Since there are no stLFR co-barcoding datasets publicly available for these model organisms, we simulated stLFR data *(*[*https://github.com/BGI-Qingdao/stLFR_reads_sim*](https://github.com/BGI-Qingdao/stLFR_reads_sim)) based on their reference assemblies (*E. coli*: [*https://ftp.ncbi.nlm.nih.gov/genomes/all/GCF/000/005/845/GCF_000005845.2_ASM584v2/GCF_000005845.2_ASM584v2_cds_from_genomic.fna.gz*](https://ftp.ncbi.nlm.nih.gov/genomes/all/GCF/000/005/845/GCF_000005845.2_ASM584v2/GCF_000005845.2_ASM584v2_cds_from_genomic.fna.gz), *S. cerevisiae*: [*https://ftp.ncbi.nlm.nih.gov/genomes/all/GCA/004/328/465/GCA_004328465.1_ASM432846v1/GCA_004328465.1_ASM432846v1_genomic.fna.gz*](https://ftp.ncbi.nlm.nih.gov/genomes/all/GCA/004/328/465/GCA_004328465.1_ASM432846v1/GCA_004328465.1_ASM432846v1_genomic.fna.gz), *C. elegans*: [*https://ftp.ncbi.nlm.nih.gov/genomes/all/GCA/000/975/215/GCA_000975215.1_Cael_CB4856_1.0/GCA_000975215.1_Cael_CB4856_1.0_genomic.fna.gz*](https://ftp.ncbi.nlm.nih.gov/genomes/all/GCA/000/975/215/GCA_000975215.1_Cael_CB4856_1.0/GCA_000975215.1_Cael_CB4856_1.0_genomic.fna.gz), and *A. thaliana*: [*https://ftp.ncbi.nlm.nih.gov/genomes/all/GCA/902/460/315/GCA_902460315.1_Arabidopsis_thaliana_Eri-1/GCA_902460315.1_Arabidopsis_thaliana_Eri-1_genomic.fna.gz*](https://ftp.ncbi.nlm.nih.gov/genomes/all/GCA/902/460/315/GCA_902460315.1_Arabidopsis_thaliana_Eri-1/GCA_902460315.1_Arabidopsis_thaliana_Eri-1_genomic.fna.gz)). The simulator was designed to generate stLFR co-barcoding datasets according to an empirical stLFR dataset model (including insert size profiling of paired-end fragments and length profiling of long DNA fragments). The datasets with 100× depth were simulated and used in our tests for all four organisms.

The contigs for *E. coli* and *C. elegans* were assembled by MaSuRCA, while contigs for *S. cerevisiae* and *A. thaliana* were assembled by SOAP*denovo2*. The simulator did not consider the effect of read errors (sequencing) and barcode collisions, and thus high-quality contigs with few misassemblies were generated as listed in Table S7 with the simulated stLFR datasets. All the four sets of contigs were next scaffolded with default parameters, and the evaluations of the scaffolding results are also listed in Table S7. Assembly improvement was evident for all the four model genomes. In the above species order, the NG50s dramatically increased – from 130 kb to 2,649 kb, 38 kb to 778 kb, 93 kb to 1,729 kb, and 23 kb to 7,334 kb. The total assembled lengths remain almost the same, but the number of scaffolds is reduced. The scaffolding results are highly accurate, as the NGA50s based on the alignments against the reference assembly also improved by 7, 18, 4, and 240 fold.
